# Identification of biomarkers and immune microenvironment associated with heart failure through bioinformatics and machine learning

**DOI:** 10.3389/fmolb.2025.1580880

**Published:** 2025-05-08

**Authors:** Jingyun Jin, Shuyan Qin, Qiang Fu, Changzhi Yu, Hongjin Wu

**Affiliations:** ^1^ School of Integrative Medicine, Shanghai University of Traditional Chinese Medicine, Shanghai, China; ^2^ Department of Preventive Treatment of Disease, Nanyang Second General Hospital, Nanyang, China; ^3^ Department of Traditional Chinese Medicine, Fuwai Hospital Chinese Academy of Medical Sciences, Shenzhen, China; ^4^ Department of Cardiovascular Medicine, Beijing Haidian Hospital, Haidian Section of Peking University Third Hospital, Beijing, China

**Keywords:** heart failure, biomarkers, bioinformatics, weighted gene co-expression network analysis, machine learning, immune infiltration

## Abstract

**Background:**

Heart failure (HF) is the end stage of various cardiovascular diseases. Identifying new biomarkers is essential for early diagnosis, prognosis, and treatment. This study applied bioinformatics to identify potential HF biomarkers and explore the role of the immune microenvironment.

**Methods:**

Gene expression data were obtained from the Gene Expression Omnibus (GEO) database. Differential expression analysis and Weighted Gene Co-expression Network Analysis (WGCNA) were used to identify key genes. Gene Ontology (GO), Kyoto Encyclopedia of Genes and Genomes (KEGG), and Gene Set Enrichment Analysis were performed. Feature genes were further determined using two machine learning algorithms, Random Forest (RF) and Least Absolute Shrinkage and Selection Operator (LASSO), with diagnostic accuracy assessed via Receiver Operating Characteristic (ROC) curves and nomograms to screen hub genes, and external datasets further were used for validation. Quantitative reverse transcription polymerase chain reaction (RT-qPCR) was used to validate the expression levels of hub genes in clinical samples. Single Sample Gene Set Enrichment Analysis and CIBERSORT algorithm were applied to evaluate immune cell infiltration in HF and its relationship with hub genes.

**Results:**

Differential analysis identified 165 differentially expressed genes (DEGs), and WGCNA revealed the “blue” module showing a significant correlation with HF. Integration of the DEGs and the “blue” module genes identified 28 common genes. KEGG pathway enrichment analysis suggested that these genes may be involved in the cytoskeleton in muscle cells pathway. Lasso and RF algorithms confirmed 7 key genes as potential biomarkers for HF, and further analysis using the ROC curve identified 4 hub genes with good diagnostic value, namely, High mobility group N 2 (*HMGN2*), Myosin Heavy Chain 6 (*MYH6*), High temperature requirement A1 (*HTRA1*), and Microfibrillar-associated protein 4 (*MFAP4*), which were validated in an external dataset and by RT-qPCR. Immune infiltration analysis revealed significant infiltration of immune cells in HF. T cells, NK cells, monocytes, and M2 macrophages play important roles in the development of HF, and the hub genes were closely associated with multiple immune cell types.

**Conclusion:**

This study identifies *HMGN2*, *HTRA1*, *MFAP4*, and *MYH6* as novel diagnostic biomarkers and potential therapeutic targets for HF. These genes are closely related to the immune microenvironment, providing new insights into the early diagnosis, treatment, and mechanistic exploration of HF.

## 1 Introduction

Heart failure (HF) is a chronic and complex clinical syndrome resulting from myocardial damage or dysfunction, characterized by a reduced ability of the heart to pump blood, which fails to meet the metabolic demands of body tissues (McDonagh et al., 2021). This leads to a range of symptoms, including shortness of breath, edema, and fatigue. As one of the main manifestations of end-stage heart disease, the incidence and mortality of HF have been steadily increasing, particularly among the elderly, who account for 80% of all HF patients (Triposkiadis et al., 2022). Current epidemiological data estimate a global prevalence of 1%–3%, affecting over 56 million individuals, with projections indicating a 46% increase by 2030. This growing burden imposes substantial economic and resource pressures on society and healthcare systems, resulting in reduced quality of life, frequent hospitalizations, increased healthcare costs, and high premature mortality rates (Virani et al., 2021; Yan et al., 2023; Bui et al., 2011). Despite significant advancements in clinical treatments—including pharmacotherapy, implantable devices, and surgical interventions that have improved survival rates and quality of life for HF patients, the 5-year survival rate after diagnosis remains below 50%, which is worse than that of certain malignant cancers (Jones et al., 2019; Mamas et al., 2017). Emerging evidence underscores the critical role of immune dysregulation in HF progression. Chronic inflammation and immune cell infiltration (e.g., T lymphocytes, macrophages) have been implicated in myocardial remodeling, fibrosis, and ventricular dysfunction (Zhang et al., 2017; Wrigley et al., 2011). However, the interplay between immune microenvironment dynamics and molecular biomarkers remains poorly characterized.

Early diagnosis of HF continues to be a major challenge. Currently available biomarkers, such as brain natriuretic peptide (*BNP*) and N-terminal pro-brain natriuretic peptide (*NT-proBNP*), play an important role in clinical practice. However, their sensitivity and specificity have certain limitations, making them insufficient for precise differentiation across HF subtypes or disease stages (Panagopoulou et al., 2013; Don-Wauchope and McKelvie, 2015). While previous studies have identified candidate biomarkers through single-omics approaches (e.g., transcriptomics or proteomics) (Guo et al., 2020; Kurniawan et al., 2024), these efforts often rely on conventional statistical methods that prioritize individual gene-level associations, overlooking network-level interactions and immune microenvironment dynamics. To address these gaps, we integrate multi-omics data with computational frameworks to prioritize robust biomarkers while elucidating their immune-pathological relevance. In recent years, the development of omics technologies, such as genomics, transcriptomics, and proteomics, alongside advancements in bioinformatics, has introduced novel strategies for identifying biomarkers based on high-throughput data. Meanwhile, machine learning algorithms have become increasingly mature in the biomedical field, optimizing data features and enhancing the accuracy and robustness of predictive models. Integrating bioinformatics analyses with machine learning models facilitates the identification of biomarkers with higher diagnostic and prognostic value, providing new perspectives for the early diagnosis, treatment, and management of HF (Xu et al., 2023; Wang et al., 2023; Zhu et al., 2023). Researchers now see immune system malfunctions as vital components of HF pathology which resembles their impact on cancer treatment resistance. PD-1 immune checkpoint molecules control T cell performance in oncology which affects both tumor progression and therapeutic responses. Heart failure disease severity and therapeutic response can be affected by immune system changes that cause T cell exhaustion and ongoing inflammation (Mustafa et al., 2024).

**FIGURE 1:**
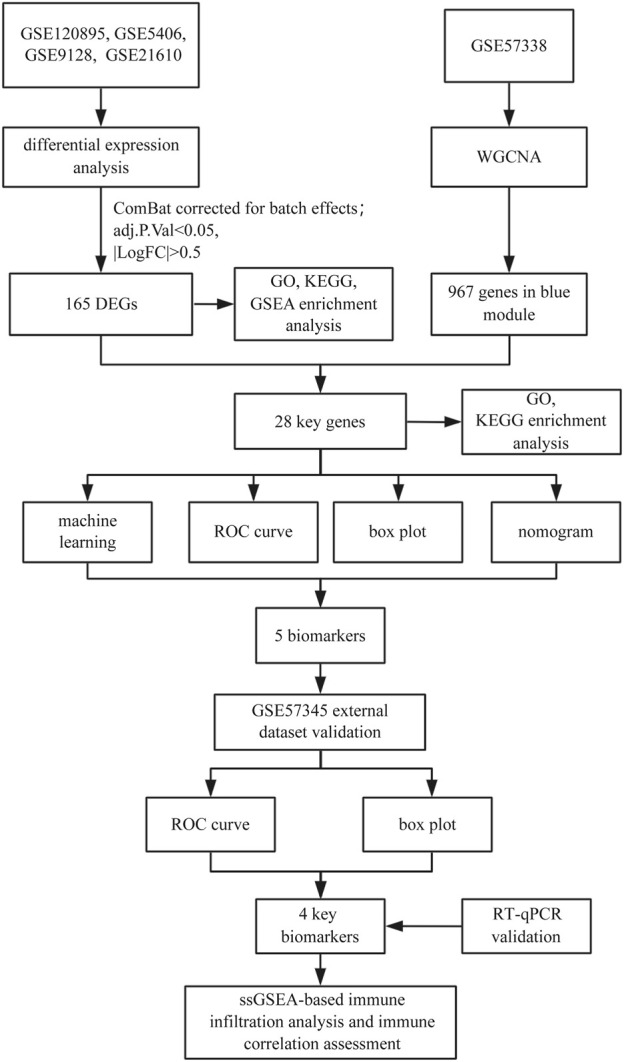
The study flowchart.

Based on this, the present study aims to integrate gene expression data from the Gene Expression Omnibus (GEO) using Weighted Gene Co-expression Network Analysis (WGCNA) and machine learning algorithms to identify potential immune-related HF biomarkers. Crucially, we further employ single-sample gene set enrichment analysis (ssGSEA) to quantify immune cell infiltration and elucidate its correlation with candidate biomarkers, thereby bridging molecular signatures and immune pathophysiology. This research seeks to provide innovative theoretical support for clinical practice in HF management. Figure 1 illustrates the study workflow.

## 2 Materials and methods

### 2.1 Data processing and differential analysis

Gene expression data for heart failure were obtained from the GEO database by searching with the keyword “heart failure”. Only datasets that met specific criteria were included in the study: 1) Species: *Homo sapiens*; 2) Data: Expression profiling by array; 3) Sample: each dataset contained ≥10 samples. Four datasets (GSE5406, GSE9128, GSE120895, and GSE21610) along with their corresponding platform annotation files were downloaded. The Perl programming language was used to annotate the data with official gene symbols and to group the samples. The R package “limma” was used with the “normalizeBetweenArrays” function to normalize the raw count expression data. Batch effects across the four datasets were removed using the “ComBat” function from the “sva” package. Differential expression analysis was performed using the Bayesian multiple testing correction method from the “limma” and “Bioconductor” packages, with the cutoff criteria for differentially expressed genes (DEGs) set as adj.P.Val <0.05 and |LogFC| > 0.5. The volcano plot was generated using the R package ggplot2, while clustering heatmaps of the top 50 upregulated and top 50 downregulated DEGs were created using the pheatmap package.

### 2.2 Gene functional enrichment analysis

Gene Ontology (GO) functional enrichment analysis includes three components: biological process (BP), cellular component (CC), and molecular function (MF). The correlation between genes and biological functions is explored by identifying the main enriched GO items (Ashburner et al., 2000). The Kyoto Encyclopedia of Genes and Genomes (KEGG) is a knowledge base for analyzing gene functions through systematic studies of gene and molecular networks. It explores the enrichment of genes in pathways such as cellular metabolism, signal transduction, and the cell cycle (Ogata et al., 1999). Gene Set Enrichment Analysis (GSEA) is a computational method based on molecular marker databases to interpret gene expression data. It is commonly used to analyze and explain pathway-level changes between normal and disease groups (Subramanian et al., 2005). The immune-related gene sets were downloaded from the Molecular Signatures Database (MSigDB) as the reference gene set, namely, “immunesigdb.gmt” file. Using the R package “org.HS.e.g.,.db”, “clusterProfiler”, DEGs were analyzed for GO, KEGG and GSEA enrichment. Enrichment results with p ≤ 0.05 and q ≤ 0.05 were considered statistically significant. Visualization of the enrichment results was performed using R packages such as “enrichplot”, “ggplot2″, “circlize”, and “ComplexHeatmap”.

### 2.3 Weighted gene co-expression network analysis

WGCNA is a systems biology method used to describe gene association patterns across different samples. WGCNA can be employed to identify clusters (modules) of highly correlated genes, summarize these clusters using module eigengenes or intramodular hub genes, correlate modules with each other and with external sample traits (using eigengene network methodology), and calculate module membership measures. Based on the interconnectivity of gene sets and their associations with phenotypes, this approach can be utilized to identify candidate biomarkers or therapeutic targets (Langfelder and Horvath, 2008). Therefore, WGCNA analysis serves as a powerful complement to DEG analysis, providing a more comprehensive perspective on pathogenic gene profiles. The GSE57345 dataset and platform annotation file were downloaded using the “getGEO” function in R. The “goodSamplesGenes” function from the “WGCNA” package was used to check for missing values in the gene expression data, and genes below the weight threshold were removed. The top 5,000 genes by average expression were selected for further analysis. Sample clustering was performed on the gene expression matrix to remove outlier samples. The “pickSoftThreshold” function was used to determine the soft threshold, and the “scaleFreePlot” function was employed to plot the scale-free distribution and the fitting line to evaluate whether the network exhibited scale-free topology. Based on the optimal soft threshold and average connectivity, the “blockwiseModules” and “plotDendroAndColors” functions from the “WGCNA” package were used to construct a gene co-expression network, identify gene modules, and plot the gene clustering dendrogram. The minimum gene number within a module was set to 50, and the module merging threshold was set to 0.5. Then, the module eigengene (ME) for each module was calculated, and the correlation between the ME and sample traits was assessed. The linear correlation coefficient (cor (ME, dataTraits)) between each module’s ME and corresponding sample traits was computed, and modules with statistically significant p-values were selected for further analysis. Based on the soft threshold, the “TOMsimilarityFromExpr” function was used to obtain topological overlap matrix (TOM). A random selection of 400 genes was made, and the topological overlap heatmap was plotted based on the TOM-based dissimilarity measure.

### 2.4 Machine learning based feature gene screening

Genes associated with both DEGs and WGCNA were intersected for feature selection. The Least Absolute Shrinkage and Selection Operator (LASSO) regression model was constructed using the “glmnet” function, and the LASSO feature gene set that minimizes the error was obtained (Friedman et al., 2010). Random forest (RF) analysis was performed using the “randomForest” function, with cross-validation error used to determine the optimal number of trees (Hu and Szymczak, 2023). The “importance” function was used to calculate and rank the importance of genes, with a threshold of importance score >2 used to identify feature genes. The intersection of the results from the two algorithms was taken to obtain the final set of feature genes.

### 2.5 The assessment of biomarkers prediction model and validation

We first performed differential expression analysis with the “limma” package and then generated box plots with the “ggpubr” package to visually depict the differences in gene expression across groups. Receiver operating characteristic (ROC) is a method used to assess the performance of classification models, and area under the curve (AUC) is commonly used as a metric to evaluate model performance. The value of AUC ranges from 0 to 1, with higher values indicating better performance. The “pROC” function was used to plot the ROC curve of feature genes and calculate the AUC value. The construction of a nomogram provides valuable reference for the diagnosis and prognosis of clinical HF. The “rms” and “regplot” functions were used to plot the nomogram and the calibration curve of the model to assess the model’s predictive performance. Finally, to validate the model’s generalizability, we used the external validation dataset GSE57345 and re-evaluated their expression level and diagnostic value through box plots and ROC curves. Hub genes were selected from the training set and validation set using the criterion of AUC >0.8.

### 2.6 Immune cell infiltration analysis

Gene set variation analysis (GSVA) is a method used to analyze gene sets and assess the variation of gene sets in samples. Single sample gene set enrichment analysis (ssGSEA) is a variation of GSVA, used to evaluate the enrichment level of specific gene sets in a given sample. The “GSVA” function was used to perform ssGSEA analysis based on gene sets for 28 immune-related cell types, evaluating the infiltration levels of immune cells in different samples. To further validate immune infiltration patterns, we applied the CIBERSORT algorithm via the ‘CIBERSORT’ R package. This method used to evaluate the percentage and abundance of 22 immune cells in tissues or cells. The “pheatmap” and “vioplot” functions were employed to generate heatmaps and box plots, displaying the infiltration abundance of immune cells between the normal and HF groups. Spearman correlation analysis was performed to assess the correlation between immune cells and hub genes, and the results were visualized using a correlation plot.

### 2.7 Clinical sample collection

To validate the results, 20 blood samples from healthy subjects (CON) and 20 blood samples from HF patients (HF) were collected from clinical sources (Wei et al., 2023). The inclusion criteria for HF patients were as follows: a. meeting the diagnostic criteria for chronic heart failure outlined in the “2024 Chinese Guidelines for the Diagnosis and Treatment of Heart Failure”. b. age between 18 and 80 years c. New York Heart Association (NYHA) class equal to or greater than II; d. *NT-proBNP* levels are higher than 450 pg/mL e. hospitalized for acute heart failure exacerbation. Exclusion criteria included severe infection, significant liver or kidney dysfunction, malignancy, severe endocrine or autoimmune diseases, and mental disorders. The control group consisted of age- and sex-matched healthy individuals from the health check-up department at our hospital during the same period. Whole blood samples (5 mL per participant) were collected in EDTA anticoagulant tubes after fasting for≥8 h. Plasma was isolated by centrifugation at 3,000 × g for 10 min within 2 h of collection, aliquoted, and stored at −80°C. The research was approved by the medical ethics committee of the Nanyang Second General Hospital (No:2024-LY051-01-H01). All participants voluntarily participated in the study and provided informed consent. Supplementary Table S1 contains the clinical data of the enrolled patients.

### 2.8 RNA extraction and quantitative real-time polymerase chain reaction

RNA extraction and RT-qPCR were performed following standard protocols. Total RNA was isolated from the samples using TRIzol. RNA concentration and purity were measured using the NanoDrop® ND-1000, and RNA integrity was assessed by denaturing agarose gel electrophoresis. Then RNA was reverse transcribed into cDNA using the SuperScript™ III Reverse Transcriptase(Invitrogen). RT-qPCR was subsequently conducted with 2x PCR Master Mix (Arraystar). Primer sequences for PCR are listed in Table 1. Each experiment was conducted in triplicate, and relative gene expression levels were calculated using the 2^–△△Ct^ method and normalized to cel-miR-39.

**TABLE 1:** Primer sequences.

Primer	Forward (5′-3′)	Reverse (5′-3′)
*HMGN2*	TGCTAAACCTGCTCCTCCAA	CTGTGCCTGGTCTGTTTTGG
*MYH6*	GCCCTTTGACATTCGCACTG	GGTTTCAGCAATGACCTTGCC
*HTRA1*	TCCCAACAGTTTGCGCCATAA	CCGGCACCTCTCGTTTAGAAA
*MFAP4*	TACCAGTCAGACGGCGTGTA	CCACTCGCAGCTCATACTTCT
*Cel-miR-39*	TCACCGGGTGTAAATCAGCTTG	TGGTGTCGTGGAGTCG

### 2.9 Statistical analysis

All bioinformatics analyses were performed using R language. Statistical analysis was conducted using GraphPad Prism 8.0.2 software. Correlations were assessed using Pearson’s correlation or Spearman’s correlation test, with statistical significance defined as a p-value less than 0.05.

## 3 Results

### 3.1 Identification of DEGs related to HF

**FIGURE 2:**
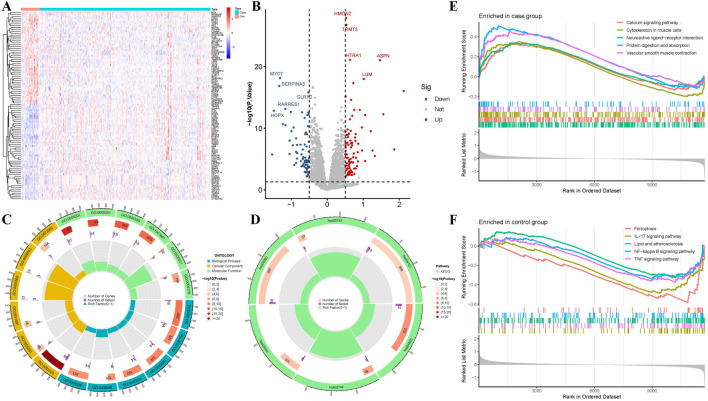
Differential analysis based on the GEO database. **(A)** Heatmap of DEGs (DEGs). **(B)** Volcano plot of DEGs. **(C)** GO enrichment analysis of DEGs. **(D)** KEGG enrichment analysis of DEGs. **(E)** GSEA analysis for the HF group. **(F)** GSEA analysis for the control group.

Four datasets (GSE5406, GSE9128, GSE120895, and GSE21610) were downloaded from the GEO database, including their expression matrix files and corresponding platform annotation files (Supplementary Table S2). A total of 165 DEGs were identified (Supplementary Table S3). Volcano plots and clustering heatmaps of the top 50 upregulated and top 50 downregulated genes were generated using R (Figures 2A, B).

### 3.2 Enrichment analysis

GO, KEGG, and GSEA functional enrichment analyses were performed on the DEGs, and the results were visualized (Figures 2C–F). The GO functional enrichment analysis identified 164 BP terms, including muscle system processes, extracellular matrix organization, regulation of leukocyte chemotaxis, muscle contraction. It also revealed 24 CC terms, such as collagen-containing extracellular matrix, collagen trimer, fibrillar collagen trimer, and banded collagen fibril, and 11 MF terms, including extracellular matrix structural constituent, heparin binding, integrin binding, collagen binding, and growth factor binding. Additionally, KEGG pathway enrichment analysis identified six pathways, including cytoskeleton in muscle cells, AGE-RAGE signaling pathway in diabetic complications, cytokine-cytokine receptor interaction, and PI3K-Akt signaling pathway.

Additionally, GSEA enrichment analysis identified 57 pathways (Figures 2E, F). Among them, 22 pathways were highly expressed in the HF group, including the calcium signaling pathway, cytoskeleton in muscle cells, Vascular smooth muscle contraction, and the renin-angiotensin system. In contrast, 35 pathways were highly expressed in the control group, such as the PI3K-Akt signaling pathway, MAPK signaling pathway, lipid and atherosclerosis, TNF signaling pathway, HIF-1 signaling pathway, and NF-kappa B signaling pathway.

### 3.3 WGCNA

**FIGURE 3:**
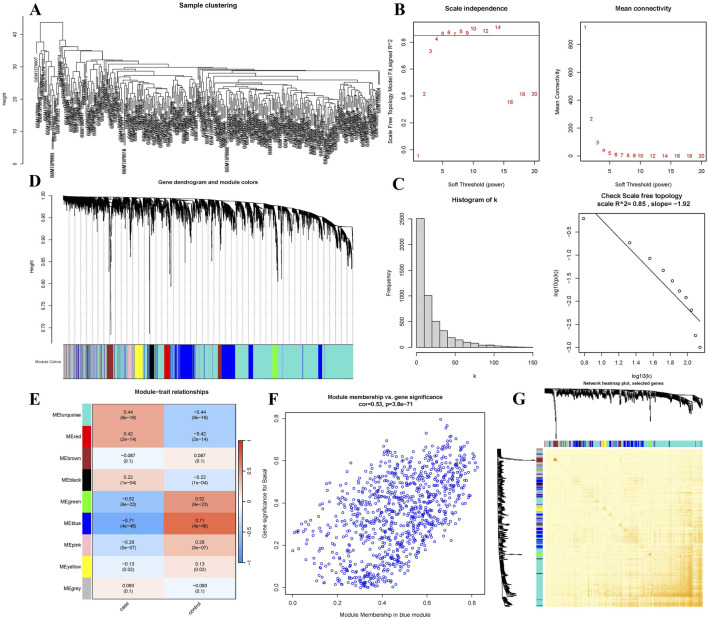
WGCNA-based analysis. **(A)** Sample clustering after removing outlier samples. **(B)** Scatter plot of soft-thresholding analysis. **(C)** Scale-free topology plot with a soft-threshold power of 5. **(D)** Gene co-expression network, with each color representing a distinct gene module. **(E)** Heatmap showing the correlation between MEs and clinical traits; darker colors indicate higher correlations. **(F)** The blue module. **(G)** TOM heatmap for a subset of genes in the blue module; lighter colors indicate lower overlap, while darker colors indicate higher overlap.

The GSE57338 dataset and its platform annotation files were obtained using R. Gene annotation was performed to derive gene expression data. Due to the large dataset size, the top 5,000 genes were selected based on their average expression values. Outlier samples GSM1379815 and GSM1380018 were removed, and the gene expression matrix was re-clustered (Figure 3A). A scale-free topology fit index *R* ^2^ of 0.85 was set, and the soft-threshold power was determined to be 5. The scatter plot indicates that beyond a power value of 5, the trend becomes stable with minimal changes (Figure 3B). To assess whether the network exhibits a scale-free topology, a scale-free topology plot and fitted line were generated, showing a linear relationship between the logarithm of the mean and the logarithm of frequency (Figure 3C).

Using the determined soft-threshold power, a gene co-expression network was constructed with the blockwiseModules function (Figure 3D). The minimum module size was set to 50 genes, and the module merging threshold was set to 0.5. After clustering, nine distinct modules were identified. To further identify gene modules significantly associated with clinical traits, correlation analysis was performed between gene modules and clinical features. The ME score for each module was calculated, and the linear correlation coefficients between the MEs and corresponding sample traits were analyzed. The results were visualized as heatmaps (Figures 3E, F). The heatmaps revealed that the blue module had the strongest correlation with heart failure, comprising 967 genes. Consequently, the blue module genes were selected for further analysis (Supplementary Table S4). Finally, a TOM was visualized by randomly selecting 400 genes to generate a TOM heatmap (Figure 3G). In the TOM heatmap, darker colors represent stronger correlations between genes.

### 3.4 Enrichment analysis

**FIGURE 4:**
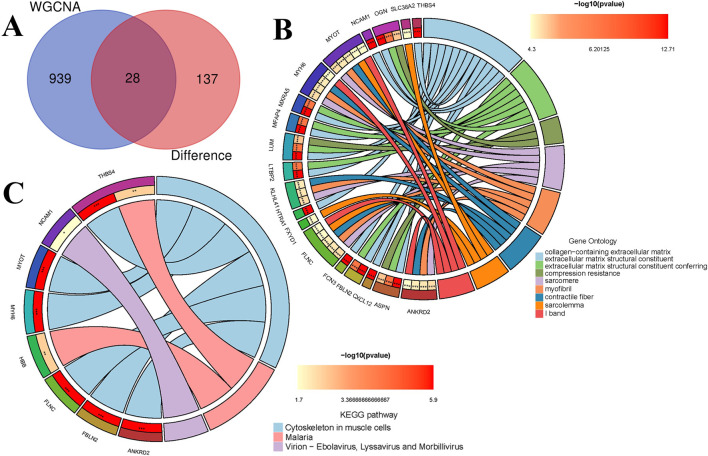
Functional enrichment analysis. **(A)** The intersection of genes obtained from WGCNA with the set of DEGs. **(B)** GO enrichment terms. **(C)** KEGG pathways.

The intersection of 165 DEGs and the 967 genes from the blue module associated with HF obtained through WGCNA resulted in 28 common genes (Figure 4A; Supplementary Table S5). The 28 common genes underwent GO and KEGG functional enrichment analyses using R (Figures 4B, C). The GO analysis identified 153 BP, mainly enriched in muscle system processes such as contraction, development, and responses to transforming growth factor β, actomyosin structure organization, complement activation. In terms of CC, 37 terms were identified, mainly enriched in collagen-containing extracellular matrix, myofibril, contractile fiber, sarcolemma and serine-type endopeptidase complex. Regarding MF, 32 terms were enriched, including extracellular matrix structural constituent, structural constituent of muscle, binding to heparin, protein kinase B, growth factor, serine transmembrane transporter activity and oxidoreductase activity. KEGG pathway analysis revealed 3 significant pathways, including cytoskeleton in muscle cells, malaria, and virion - ebolavirus, lyssavirus and morbillivirus pathway.

### 3.5 Machine learning based feature gene screening

**FIGURE 5:**
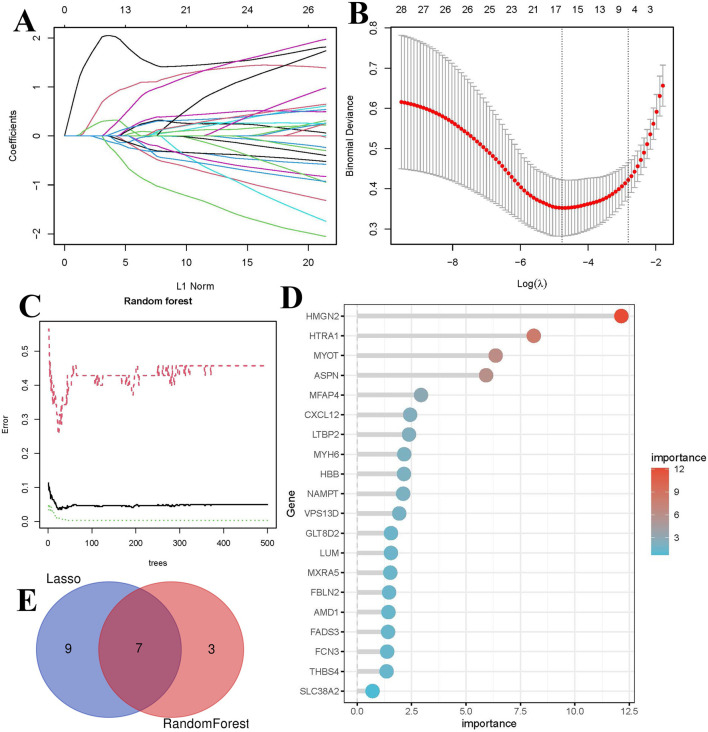
Identification of biomarkers using machine learning. **(A)** LASSO coefficient path plot, each curve represents the trajectory of a biomarker, with the vertical axis indicating gene values, the lower horizontal axis representing log(λ), and the upper horizontal axis showing the number of nonzero biomarkers in the model. **(B)** Ten-fold cross-validation plot for LASSO regression. **(C)** Relationship between the number of decision trees and error rate in the RF algorithm. **(D)** Bar plot of feature gene importance in the RF model. **(E)** Venn diagram of key biomarkers identified by both methods.

To ensure that the selected common genes reflect actual biological information as accurately as possible, two machine learning algorithms, RF and LASSO regression, were employed to further filter feature genes. LASSO regression identified 16 genes strongly associated with HF (Figures 5A, B). The RF algorithm identified 10 genes with importance scores greater than 2 (Figures 5C, D). The intersection of results from the two machine learning methods yielded seven feature genes (Figure 5E).

### 3.6 Prediction model construction and biomarkers selection

**FIGURE 6:**
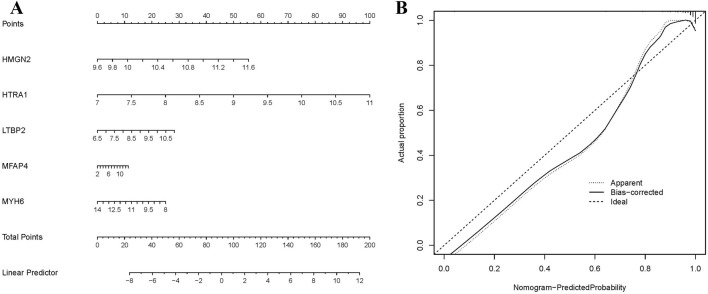
Nomogram and calibration curve of key biomarkers. **(A)** Prognostic nomogram diagram. **(B)** Calibration curve plot for the nomogram. The X-axis represents the predictable probability, and the Y-axis represents the actual probability.

**FIGURE 7:**
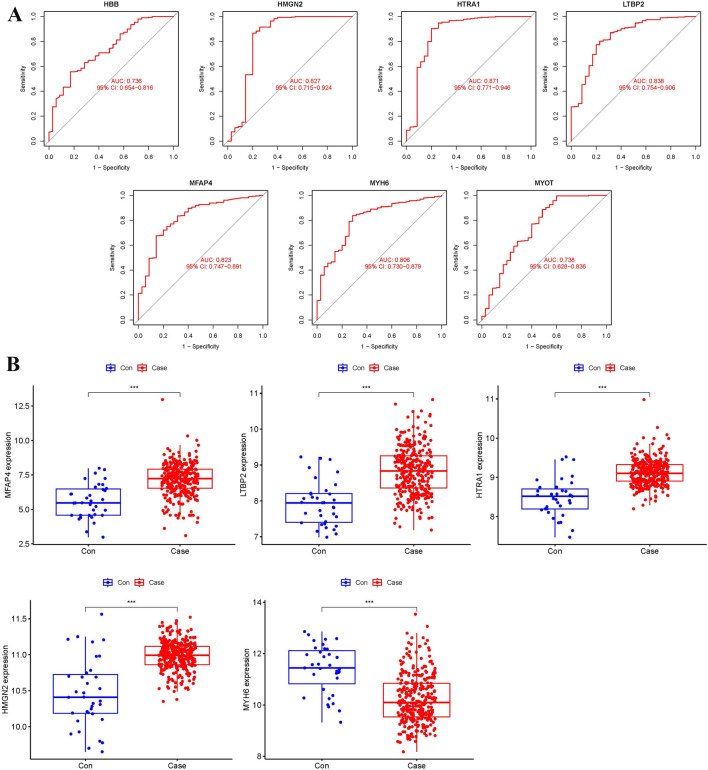
ROC curves and expression levels of biomarkers. **(A)** ROC curve analysis of biomarkers. **(B)** Box plot of biomarker expression levels in dataset samples (***p < 0.001).

To evaluate the diagnostic significance of biomarkers for HF, a nomogram and calibration curve were constructed using R (Figure 6). The calibration curve demonstrated a good fit between the predicted and actual probabilities. ROC curves were then generated to further assess the diagnostic specificity and sensitivity of the biomarkers (Figure 7A), with an AUC value >0.8 considered indicative of excellent diagnostic performance. The ROC analysis identified five biomarkers with AUC values >0.8, namely, High mobility group N 2 (*HMGN2*), Myosin heavy chain 6 (*MYH6*), High temperature requirement A1 (*HTRA1*), Latent transforming growth factor beta binding protein 2 (*LTBP2*) and Microfibrillar-associated protein 4 (*MFAP4*).

To further validate the expression levels of the five biomarkers in HF, differential expression analysis was performed on the dataset samples. The results showed that, compared to normal samples, *HMGN2*, *HTRA1*, *LTBP2*, and *MFAP4* were significantly upregulated in heart failure, while *MYH6* was downregulated (p < 0.001) (Figure 7B).

### 3.7 External validation of the key biomarkers

**FIGURE 8:**
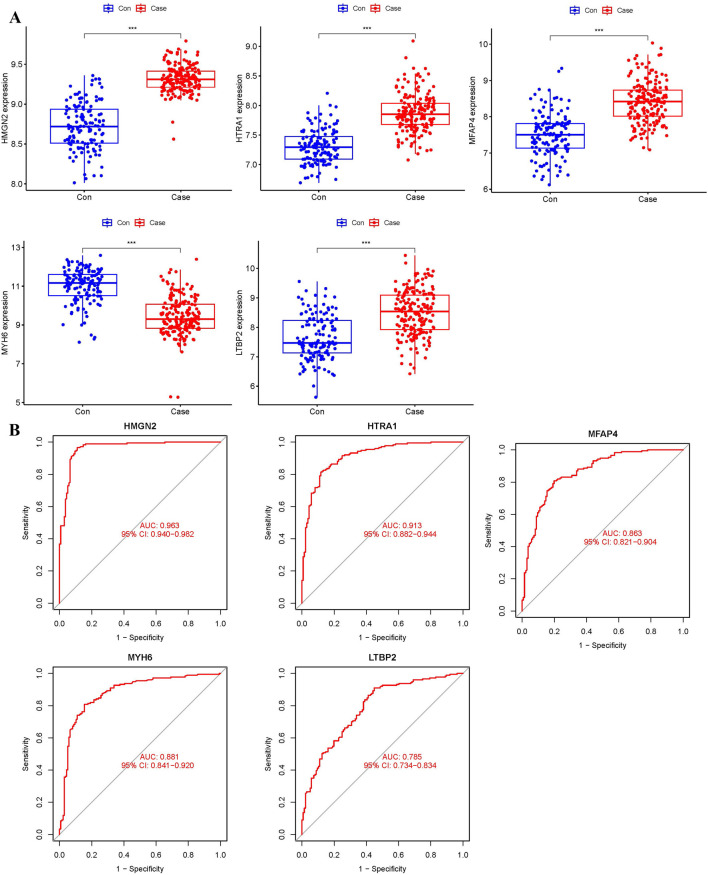
ROC curves and expression levels of biomarkers validated using the external dataset GSE57345. **(A)** Box plot of biomarker expression levels (***p < 0.001). **(B)** ROC curve analysis of biomarkers.

To ensure the accuracy of the results, an external dataset (GSE57345) was used to validate the five biomarkers. ROC curves and box plots were generated to assess their diagnostic value and expression levels (Figure 8). The results showed that the expression patterns of the five biomarkers were consistent with those in the training dataset. *HMGN2*, *HTRA1*, *MFAP4*, and *MYH6* exhibited AUC values greater than 0.8, indicating high diagnostic performance, whereas *LTBP2* had an AUC value of 0.785, suggesting moderate diagnostic accuracy. Therefore, *HMGN2*, *HTRA1*, *MFAP4*, and *MYH6* were selected as key biomarkers for further analysis and validation.

**FIGURE 9:**
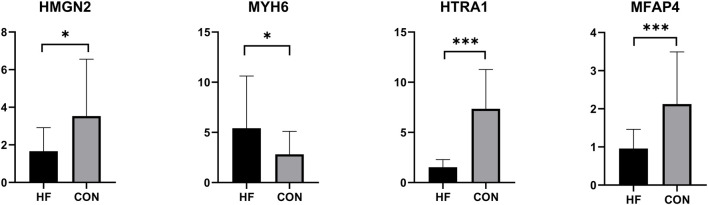
Results of RT-qPCR analysis (*p < 0.05, ***p < 0.001).

To further confirm the accuracy of the above integrated bioinformatics analysis, we analyzed the mRNA expression of the four key biomarkers in plasma samples from healthy individuals and HF patients using RT-qPCR. RT-qPCR results showed that the mRNA expression of *HMGN2*, *HTRA1*, and *MFAP4* was significantly downregulated in the plasma of HF patients compared to the control group, while *MYH6* was significantly upregulated (Figure 9; Supplementary Table S6).

### 3.8 Immune cell infiltration and its correlation with key biomarkers

**FIGURE 10:**
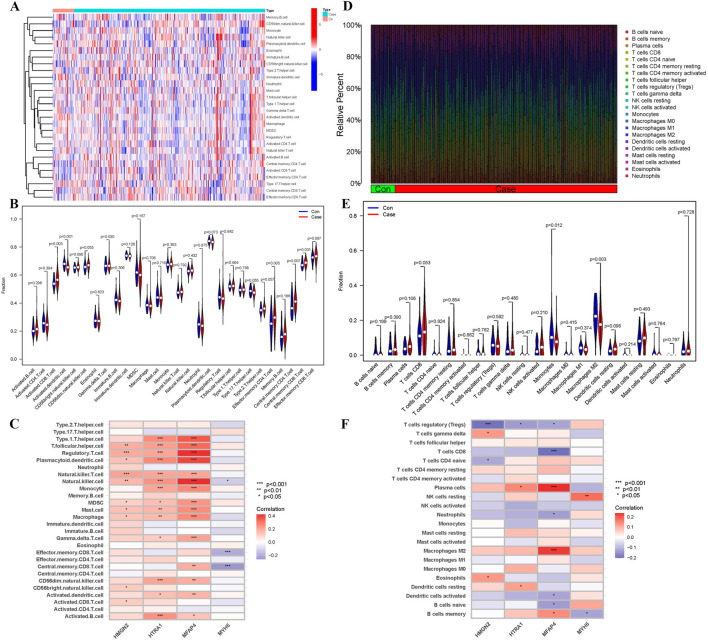
Immune infiltration analysis. **(A)** Heatmap of immune scores for 28 immune cell types in ssGSEA, with the x-axis representing sample names, the y-axis representing different immune cells, and the clustering tree on the left indicating the clustering of units on the vertical axis. Red represents immune cell infiltration, while blue indicates immune cell suppression. The intensity of the color represents the degree of cell infiltration. **(B)** Boxplot of 28 immune cells abundance. Blue represents control and red represents heart failure. **(C)** The correlation heat map between key biomarkers and immune cells in ssGSEA. Red represents positive correlation and blue represents negative correlation. **(D)** Bar graph of 22 immune cells percentages in CIBERSORT. Horizontal coordinates represent samples, vertical coordinates represent percentages, and colors represent immune cells. **(E)** Boxplot of 22 immune cells abundance in CIBERSORT. **(F)** The correlation heat map between key biomarkers and immune cells in CIBERSORT (*p < 0.05, **p < 0.01, ***p < 0.001).

To further investigate the immune status differences between HF patients and healthy controls, immune infiltration analysis was performed by ssGSEA and CIBERSORT algorithms. Figure 10A illustrates the distribution of 28 immune cell types in the dataset based on ssGSEA. Among 28 immune cells, the infiltration rates of Activated CD8^+^ T cells, Effector memory CD4^+^ T cells, Central memory CD4^+^ T cells, and Central memory CD8^+^ T cells were significantly higher in HF samples than in normal samples. In contrast, the infiltration rate of Activated dendritic cells was significantly lower in HF samples (Figure 10B). Correlation analysis between key genes and immune cells revealed that *HMGN2, HTRA1*, and *MFAP4* were positively correlated with T follicular helper cells, regulatory T cells, plasmacytoid dendritic cells, natural killer T cells, monocytes, myeloid-derived suppressor cells, mast cells, macrophages, CD56 dim natural killer cell, activated dendritic cells, and activated B cells. In contrast, *MYH6* was negatively correlated with effector memory CD8^+^ T cells and central memory CD8^+^ T cells (Figure 10C).

Histogram showed the composition of 22 different immune cell types in each sample based on the CIBERSORT (Figure 10D). The color representation corresponds to the percentage of each immune cell type in each sample, with the total sum equaling 1. The analysis results indicated that T cells and NK cells occupy a larger proportion. Among 22 immune cells, the HF samples were associated with significantly decreased abundances of Monocytes and Macrophages M2 (Figure 10E). Correlation analysis between key genes and immune cells revealed that *HMGN2* was positively associated with T cell gamma delta and Eosinophils, and negatively associated with T cells regulatory (Tregs) and T cells CD8. *HTRA1* was positively associated with Plasma cells and Dendritic cells resting, and negatively associated with T cells regulatory (Tregs). *MFAP4* was positively associated with Plasma cells, Macrophages M2 and B cells memory,and negatively associated with T cell CD8, T cells regulatory (Tregs), Neutrophils, Dendritic cells activated, B cells naive. *MYH6* was positively associated with NK cells resting, and negatively associated with B cells memory (Figure 10F). These findings further support the regulatory role of immune cells in the molecular mechanisms underlying heart failure progression.

## 4 Discussion

Heart failure is a severe cardiac condition whose seriousness and prevalence pose a significant challenge to global public health. Currently, the treatment of HF primarily focuses on alleviating symptoms and slowing disease progression, yet there is a lack of effective early diagnostic methods and targeted therapies. In clinical practice, various biomarkers have been utilized for the diagnosis and prognosis assessment of heart failure, such as *BNP*, *NT-proBNP*, *cTn*, galectin-3, *sST2*, and growth differentiation factor-15. However, their clinical application remains limited by issues related to sensitivity, specificity, and their ability to identify early-stage populations (Wang et al., 2017; Wang et al., 2018). For instance, a systematic review showed that BNP exhibits a sensitivity ranging from 91% to 95% but a specificity limited to 55%–80%, while NT-proBNP demonstrates a sensitivity of 90%–96% and a specificity of approximately 55%–74%, which may still be influenced by factors such as age and renal dysfunction (Hill et al., 2014). In this study, we conducted bioinformatics analyses on public databases and validated the findings using blood samples from heart failure patients. We identified four diagnostic biomarkers for HF, namely, *HMGN2*, *HTRA1*, *MFAP4*, and *MYH6*, which demonstrated diagnostic performance comparable to conventional biomarkers (AUC>0.8) in external validation. Furthermore, unlike traditional biomarkers, the association of these genes with immune microenvironment dynamics may provide novel insights into molecular subtyping of HF, offering new perspectives for future research.

*HMGN* proteins are a class of non-histone chromatin architectural proteins located in the nucleus and exclusively expressed in eukaryotes, playing roles in regulating transcription and DNA repair (Murphy et al., 2017). As a member of the *HMGN* family, *HMGN2* is a key regulator of transcriptional activation in gene expression and has been shown to significantly inhibit tumor cell proliferation, migration, and angiogenesis, exerting anti-tumor effects (Fan et al., 2019; Xu et al., 2020). The role of *HMGN2* in HF has not been fully elucidated, but studies suggest that endogenous *HMGN2* acts as a positive regulator of NF-κB signaling and modulates intracellular ROS homeostasis through the Nrf2 pathway, thereby regulating oxidative stress and actin cytoskeleton rearrangement (Liu et al., 2017). These findings imply that *HMGN2* may influence myocardial cell homeostasis and stress responses by participating in chromatin structure regulation, gene transcription, and oxidative stress, thereby impacting HF. Moreover, previous research has identified *HMGN2* as one of the HF signature genes (Li et al., 2020), which is consistent with our results. Validation using external datasets and analyses revealed that *HMGN2* is highly expressed in myocardial tissues of HF patients, while RT-qPCR results indicated low expression of *HMGN2* in the plasma of HF patients. This discrepancy may be attributed to the following reasons: HF-related oxidative stress and inflammation may upregulate *HMGN2* expression in myocardial tissues to protect cardiomyocytes from damage; mechanical stress induced by HF may promote *HMGN2*-mediated actin rearrangement to adapt to changes in cardiomyocyte morphology and function, leading to its high expression in myocardial tissues. The low expression of *HMGN2* in the plasma of HF patients could be explained by several factors: *HMGN2* primarily functions as a chromatin regulatory protein within the nucleus, modulating gene transcription and thus is less likely to be released into the bloodstream, remaining localized in myocardial tissues to regulate local antioxidant stress and autophagy; in HF, changes in the vascular microenvironment may affect *HMGN2* release, such as reduced secretion by smooth muscle cells and endothelial cells, thereby lowering *HMGN2* levels in the blood; *HMGN2* in plasma may be rapidly degraded or cleared.

*HTRA1* is a member of the *HTRA* family and a serine protease involved in critical biological processes such as cell proliferation, mitochondrial homeostasis, and apoptosis. Abnormalities in its structure and function can influence the expression of transforming growth factor-beta (*TGF-β*), thereby affecting the progression of cardiovascular diseases (Zhao et al., 2023; Fasano et al., 2020). Studies have shown that *HTRA1* is significantly elevated in Dilated cardiomyopathy (DCM), and its inhibition can effectively prevent the transformation of cardiac fibroblasts into myofibroblasts, thereby significantly suppressing myocardial fibrosis and improving left ventricular function in DCM mice (Shi et al., 2024). These findings suggest that HTRA1 may reduce TGF-β-mediated fibroblast activation and collagen production by modulating TGF-β activity, thereby suppressing myocardial fibrosis and improving left ventricular function. A study found that increased circulating *HTRA1* levels are causally associated with a reduced risk of coronary artery disease. The mechanism may involve increased *HTRA1* expression in smooth muscle cells and endothelial cells, which inhibits TGF-beta signaling in atherosclerosis, thereby preventing neointima formation and pathological endothelial-mesenchymal transition. *HTRA1* was identified as a potential therapeutic target for coronary artery disease (Lee et al., 2024). Another study also highlighted the central role of *HTRA1* in coronary artery disease, discovering that a common causal variant (rs2672592) regulates circulating *HTRA1* mRNA and protein levels, increasing the risk of ischemic stroke, small vessel stroke, and coronary artery disease (Dichgans et al., 2023). Validation using external datasets and analyses revealed that *HTRA1* is highly expressed in myocardial tissues of heart failure patients, while RT-qPCR results indicated low expression of *HTRA1* in the plasma of HF patients. This discrepancy may be due to the following reasons: in HF patients, the progression of myocardial fibrosis leads to increased local enrichment of *HTRA1* in myocardial tissues rather than its release into the bloodstream, resulting in relatively lower plasma levels; *HTRA1* may be locally consumed during the myocardial fibrosis process and not sufficiently released into the circulation; or endothelial dysfunction associated with HF may affect the expression and release of *HTRA1*, with chronic oxidative stress leading to its degradation in the circulatory system.

*MFAP4* is an extracellular matrix (ECM) glycoprotein, abundantly expressed in elastin-rich tissues such as skin, arteries, lungs, and the heart (Mohammadi et al., 2022). *MFAP4* is closely associated with various remodeling-related diseases, such as atherosclerosis and arterial injury-induced remodeling. Its expression is significantly increased in heart failure animal models and *TGF-β*-stimulated cardiac fibroblasts, while the deletion of this gene can attenuate left ventricular remodeling and dysfunction in heart failure (Wang et al., 2020). A clinical cohort study revealed that *MFAP4* protein is primarily located on elastic fibers within blood vessels, with its synthesis mainly derived from vascular smooth muscle cells (VSMCs). The study also noted that serum *MFAP4* levels in patients with stable atherosclerotic disease were lower than in healthy individuals. This may be due to the release of *MFAP4* from VSMCs in the medial layer into the circulation, or the increased elastase activity in atherosclerosis, which reduces elastin content in atherosclerotic vessels, leading to decreased *MFAP4* synthesis bound to elastin in the ECM (Wulf-Johansson et al., 2013). A study has found that knockout of the *MFAP4* gene exacerbates age-related elastin/collagen ratios, leading to elastin degradation, while improving Ang II-induced diastolic hypertension by reducing the stiffness of mesenteric resistance arteries (Christensen et al., 2024). It also reduces susceptibility to AF by inhibiting the activation of the PI3K-AKT and MEK1/2-ERK1/2 signaling pathways, thereby suppressing Ang II-induced atrial fibrosis and AF progression (Wang et al., 2022). Analysis suggests that circulating *MFAP4* exhibits bidirectional changes in cardiovascular diseases, decreasing in stable atherosclerosis but increasing in ST-segment elevation myocardial infarction (STEMI) and non-STEMI patients, indicating that circulating *MFAP4* levels depend on the degree of vascular wall calcification and injury in the context of cardiovascular disease. Furthermore, the role of *MFAP4* in cardiac remodeling presents conflicting data. On one hand, *MFAP4* deficiency reverses aortic constriction and isoproterenol-induced cardiac dysfunction without affecting cardiac hypertrophy in these models (Wang et al., 2020). On the other hand, *MFAP4* is considered protective in stress-induced cardiac hypertrophy (Dorn et al., 2021a). Although some studies suggest that *MFAP4* is involved in cardiac fibrosis (Wang et al., 2020), others have found no observed effect of *MFAP4* deficiency on the development of local fibrosis (Dorn et al., 2021b). This implies that the role of *MFAP4* in cardiac remodeling may depend on cell type. In early disease stages, *MFAP4* signaling in cardiomyocytes may be beneficial, but in advanced disease, it may activate pro-fibrotic pathways in non-myocytes such as endothelial cells and cardiac fibroblasts (Kanaan et al., 2022). The application of *MFAP4* in heart failure requires further validation. External dataset validation and analyses indicate that *MFAP4* is highly expressed in myocardial tissues of heart failure patients, consistent with previous studies (Wang et al., 2020), while RT-qPCR results show low expression of *MFAP4* in the plasma of HF patients. This discrepancy may be due to significant ECM remodeling in HF patients, causing *MFAP4* to preferentially bind to the ECM rather than being released into the bloodstream; increased activity of ECM-degrading enzymes (e.g., elastase) accelerates *MFAP4* degradation; and HF-associated vascular remodeling, changes in vascular elastin structure, and vascular dysfunction may impair *MFAP4* entry into the circulation.

The *MYH6* gene encodes the α-heavy chain subunit of cardiac myosin (αMyHC), a key protein in myocardial contraction. In normal hearts, αMyHC mRNA accounts for 20%–30% of total myosin mRNA, and its protein constitutes approximately 7% of total MyHC. In heart failure, both αMyHC mRNA and protein levels are significantly downregulated to around 10% (Lv et al., 2011; Theis et al., 2015). Changes in MYH6 gene expression can lead to alterations in myocardial structure, thereby affecting ventricular remodeling, resulting in cardiac enlargement and sinus node dysfunction, which are closely associated with ischemic cardiomyopathy and HF (Chen et al., 2021). αMyHC is closely linked to the phenotypes of both DCM and hypertrophic cardiomyopathy (HCM). Studies have shown that αMyHC mRNA expression is downregulated in DCM patients, and β-blocker therapy can restore αMyHC fibers, thereby improving myocardial function (Lowes et al., 2002). Additionally, *MYH6* mutations can lead to a spectrum of dilated and hypertrophic phenotypic changes, ranging from DCM to HCM, including myocardial hypertrophy progressing to dilation and systolic dysfunction (Carniel et al., 2005), as well as severe adverse outcomes in DCM patients, such as sudden death and heart failure (Merlo et al., 2013). External dataset validation and analyses indicate that *MYH6* is lowly expressed in myocardial tissues of heart failure patients, while RT-qPCR results show that *MYH6* is highly expressed in the plasma of HF patients. This discrepancy may be due to the fact that *MYH6* is primarily an intracellular structural protein, with its expression largely confined to cardiomyocytes in healthy individuals. In the context of heart failure, cardiomyocyte damage or apoptosis may lead to the release of intracellular *MYH6* into the bloodstream, resulting in elevated plasma *MYH6* levels. Additionally, the inflammatory and immune responses accompanying heart failure may also influence the expression and release of *MYH6*.

Furthermore, KEGG pathway enrichment analysis of the hub genes revealed that they are primarily concentrated in the “Cytoskeleton in muscle cells” pathway. The cytoskeleton of cardiomyocytes is composed of actin, intermediate filament proteins such as desmin, and α- and β-tubulins, which polymerize to form microtubules. These components provide structural support, regulate cell shape, ensure mechanical integrity, and stabilize sarcomeric proteins. Additionally, the cytoskeletal framework mediates biomechanical and biochemical signaling between the intracellular and extracellular environments, influencing gene expression, post-translational regulation, and protein synthesis, ultimately leading to direct myocardial remodeling (Sequeira et al., 2014). Alterations in the cytoskeleton, particularly changes in microtubules and desmin, play a significant role in cardiac hypertrophy and heart failure. Studies have shown that in human hearts with chronic heart failure caused by DCM, the morphological basis of reduced contractile function is the disorganization and accumulation of cytoskeletal and membrane-associated proteins (Hein et al., 2000).

HF is often accompanied by complex immune responses, including the infiltration of inflammatory cells and the release of cytokines. The activation of cardiac immune response mechanisms triggers adverse cardiac remodeling and leads to left ventricular dysfunction. Understanding the molecular mechanisms by which immune responses interfere with cardiac remodeling in HF may open new avenues for designing biomarkers or drug targets (Zhang et al., 2017). The ssGSEA immune infiltration analysis revealed a higher abundance of T cells in HF compared to the control group. CIBERSORT immune infiltration analysis suggested that T cells and NK cells constituted a larger proportion, while monocytes and M2 macrophages showed significantly reduced abundance in HF. Correlation analysis of key genes with immune cells indicated that HMGN2, HTRA1, and MFAP4 might exert diverse regulatory effects on T cells and macrophages. MYH6 was found to potentially regulate both T cells and NK cells. This suggests that these immune cells play important roles in the development of HF. Research indicates that end-stage HF is characterized by the accumulation of T cells in the ventricles, and infiltration of T cells can be observed in animal models of failing hearts (Laroumanie et al., 2014). T cells activation, coupled with LV endothelial activation, promotes T-cell infiltration into the LV. This process exacerbates HF through mechanisms involving cytokine release and induction of cardiac fibrosis and hypertrophy (Nevers et al., 2015). Clinical samples further demonstrate a positive correlation between inflammatory cytokines produced by T cells and the severity of LV dysfunction in HF patients (Fukunaga et al., 2007). Experimental evidence found that in murine HF models, blockade of T-cell costimulation significantly delayed disease progression and reduced cardiac dysfunction severity. This therapeutic effect was attributed to suppressed activation and cardiac infiltration of T cells, ultimately decreasing cardiomyocyte death (Kallikourdis et al., 2017). Emerging evidence indicates that NK cells can limit cardiac inflammation and fibrosis, and ameliorate postinfarct cardiac remodeling and failure (Sobirin et al., 2012; Ong et al., 2015). Specifically, NK cells mitigate cardiac fibrosis progression through directly restricting collagen formation of cardiac fibroblasts and the accumulation of specific inflammatory populations and eosinophils in the heart (Sun et al., 2021). Macrophages, a major cell population involved in cardiac immune response and inflammation, are polarized into M1 and M2 types. M1 macrophage releases inflammatory factors and chemokines to activate the immune response, while M2 macrophage releases anti-inflammatory factors to inhibit the overactive immune response and promote tissue repair (Zhu et al., 2024). A study found that M1 macrophages were elevated, while M2 macrophages decreased in HF mice (Zhang et al., 2021). The above results are consistent with our findings.

This study has several limitations. For instance, the sample size is limited, and further expansion is needed to validate the reliability of the results. The inconsistency in the validation of gene differential expression levels may be attributed to differences between datasets and validation samples. Myocardial tissues reflect local pathological changes, while gene levels in plasma may be influenced by systemic metabolism, clearance rates, and release mechanisms, reflecting changes at different biological levels. Additionally, limitations in RT-qPCR detection, such as sample processing, RNA extraction efficiency, and the stability of circulating RNA, may contribute to inaccurate results. Future studies should focus on detecting protein levels in both myocardial tissues and plasma, as well as conducting more in-depth experimental research, such as cell or animal model experiments (e.g., knockout/overexpression models), to comprehensively evaluate expression changes and further explore the potential mechanisms of these biomarkers in the development and progression of HF. In summary, this study utilized bioinformatics methods to identify a group of potential biomarkers associated with HF. These biomarkers hold promise for providing new tools for the early diagnosis, prognosis assessment, and personalized treatment of HF. However, these findings require further experimental validation and confirmation through clinical studies.

## 5 Conclusion

In this study, we employed bioinformatics and machine learning methods to identify four potential diagnostic biomarkers for HF, namely, *HMGN2*, *HTRA1*, *MFAP4*, and *MYH6*. Using ROC curve analysis and nomogram construction, we developed diagnostic and predictive models that demonstrated excellent diagnostic performance and HF risk prediction capabilities. The expression levels of these biomarkers were further validated using blood samples from clinical patients. Finally, we applied the ssGSEA and CIBERSORT algorithm to analyze immune infiltration in HF patients, and correlation analysis revealed that the hub genes are involved in the immune response of HF. In summary, these four biomarkers may play critical roles in the development and progression of HF and hold promise for early diagnosis and prognosis assessment of HF, identifying high-risk populations, and guiding personalized treatment strategies.

## Data Availability

The datasets presented in this study can be found in online repositories. The names of the repository/repositories and accession number(s) can be found in the article/Supplementary Material.

## Abbreviations

HF, Heart failure; AUC, area under the curve; BNP, brain natriuretic peptide; BP, biological process; CC, cellular component; DEGs, differentially expressed genes; GEO, Gene Expression Omnibus; GO, Gene Ontology; GSEA, Gene Set Enrichment Analysis; GSVA, Gene Set Variation Analysis; KEGG, Kyoto Encyclopedia of Genes and Genomes; LASSO, Least Absolute Shrinkage and Selection Operator; ME, module eigengene; MF, molecular function; MSigDB, Molecular Signatures Database; NT-proBNP, N-terminal pro-brain natriuretic peptide; ROC, Receiver operating characteristic; RT-qPCR, reverse transcription polymerase chain reaction; ssGSEA, Single Sample Gene Set Enrichment Analysis; TOM, topological overlap matrix; WGCNA, Weighted Gene Co-expression Network Analysis.
